# Elevated Colonization of Microborers at a Volcanically Acidified Coral Reef

**DOI:** 10.1371/journal.pone.0159818

**Published:** 2016-07-28

**Authors:** Ian C. Enochs, Derek P. Manzello, Aline Tribollet, Lauren Valentino, Graham Kolodziej, Emily M. Donham, Mark D. Fitchett, Renee Carlton, Nichole N. Price

**Affiliations:** 1 Rosenstiel School of Marine and Atmospheric Science, University of Miami, 4600 Rickenbacker Cswy., Miami, Florida, 33149, United States of America; 2 Atlantic Oceanographic and Meteorological Laboratories (AOML), NOAA, 4301 Rickenbacker Cswy., Miami, Florida, 33149, United States of America; 3 IRD-Sorbonne Universités (Univ. Paris 6) UPMC-CNRS-MNHN, Laboratoire IPSL-LOCEAN, 32 Avenue Henri Varagnat, 93143, Bondy, France; 4 Moss Landing Marine Laboratories, 8272 Moss Landing Rd., Moss Landing, California, 95039, United States of America; 5 Bigelow Laboratory for Ocean Sciences, 60 Bigelow Dr., East Boothbay, Maine, 04544, United States of America; University of Bologna, ITALY

## Abstract

Experiments have demonstrated that ocean acidification (OA) conditions projected to occur by the end of the century will slow the calcification of numerous coral species and accelerate the biological erosion of reef habitats (bioerosion). Microborers, which bore holes less than 100 μm diameter, are one of the most pervasive agents of bioerosion and are present throughout all calcium carbonate substrates within the reef environment. The response of diverse reef functional groups to OA is known from real-world ecosystems, but to date our understanding of the relationship between ocean pH and carbonate dissolution by microborers is limited to controlled laboratory experiments. Here we examine the settlement of microborers to pure mineral calcium carbonate substrates (calcite) along a natural pH gradient at a volcanically acidified reef at Maug, Commonwealth of the Northern Mariana Islands (CNMI). Colonization of pioneer microborers was higher in the lower pH waters near the vent field. Depth of microborer penetration was highly variable both among and within sites (4.2–195.5 μm) over the short duration of the study (3 mo.) and no clear relationship to increasing CO_2_ was observed. Calculated rates of biogenic dissolution, however, were highest at the two sites closer to the vent and were not significantly different from each other. These data represent the first evidence of OA-enhancement of microboring flora colonization in newly available substrates and provide further evidence that microborers, especially bioeroding chlorophytes, respond positively to low pH. The accelerated breakdown and dissolution of reef framework structures with OA will likely lead to declines in structural complexity and integrity, as well as possible loss of essential habitat.

## Introduction

Ocean acidification (OA) describes the global oceanic uptake of atmospheric carbon dioxide, resulting in more acidic waters, with a lower saturation state (Ω) of calcium carbonate [1, 2]. The negative implications of this phenomenon are especially relevant to coral reef ecosystems which rely on calcified structures to provide habitat for diverse plants and animals [3, 4]. Coral reef habitat is maintained by a balance of constructive processes (accretion) primarily due to reef-building corals, and destructive processes (erosion), mainly due to biological erosion (bioerosion) resulting from the activity of a diverse suite of flora and fauna [5, 6].

The link between OA and habitat-forming corals is well established. Elevated pCO_2_ and reduced Ω can result in depressed calcification and growth of numerous species [7], as well as reduced fertilization success and larval settlement [8]. OA may also enhance the growth and competitive ability of fleshy macroalgae, restricting coral growth, and ultimately reducing calcification [3, 9, 10].

Hoegh-Guldberg *et al*. [11] were the first to suggest that OA may lead to the loss of reef structures and Manzello *et al*. [12] provided the first evidence suggesting that OA may accelerate bioerosion. Multiple experimental and field studies have since confirmed that OA stimulates bioerosion and abiotic carbonate dissolution, completing a two-front attack (slower calcification and faster erosion) on the production and persistence of coral reef carbonates [13–20]. Experimental evidence of OA-enhanced erosion has been restricted to taxa that employ chemical means of dissolution, rather than mechanical scraping or rasping. Clionaid sponges, which excavate complex networks and galleries from coral reef frameworks, exhibit enhanced rates of carbonate dissolution in seawater pCO_2_ conditions projected to occur by the end of the century [14, 19, 20]. On much finer scales, microborers (<100 μm) demonstrate a similar response to OA in experimentally manipulated seawater conditions [13, 15].

Despite their small size, these microbial bioeroding organisms (euendoliths *sensu* Golubic *et al*. [21]) occur in all carbonate substrates, making them important agents of bioerosion on coral reefs [5,6]. This multi-phyletic community of bioeroders consists of cyanobacteria, chlorophytes, rhodophytes, and fungi [22, 23]. Initial colonization occurs rapidly, within several days of substrate exposure, after which they continue to bore into the carbonates they occupy [22, 24]. The means by which microborers dissolve the materials in which they live is poorly understood and mechanisms have been proposed ranging from the existence of extracellular organelles [25], to the direct excretion of acid [26]. Garcia-Pichel *et al*. [27] have pointed out that, in the case of photosynthetic microboring cyanobacteria, autotrophy (i.e., the removal of CO_2_) would favor carbonate precipitation rather than dissolution but because of an active pumping of calcium ions across the cellular membrane, dissolution of CaCO_3_ is possible at the filament apex (front of dissolution) [27]. In chlorophytes, such a mechanism has presently not been shown but undersaturation at the carbonate interface and dissolution could be accomplished similarly [27]. Further research is needed among microboring taxa as different dissolution mechanisms could respond differently to environmental changes, such as OA.

Microborer filament abundances and boring rates are highly dynamic [28], dependent on numerous environmental factors, including light and sedimentation [29], nutrients [30–32], temperature and pH [13, 15], as well as various biological factors including grazing and epilithic algal cover [28, 30]. Gross biogenic dissolution rates up to one kilogram of calcium carbonate per m^2^ of reef per year have been reported from offshore sites on the Great Barrier Reef [29]. Considering the widespread occurrence of microborers in carbonate structures, both living and dead, this can add up to a large quantity of dissolved carbonate material. In some locations, this pervasiveness coupled with OA-enhanced erosion may have ramifications for carbonate budgets, contributing towards net erosional systems and the loss of reef habitat [20].

Experimental evidence of OA-enhanced microborer erosion was first presented by Tribollet *et al*. [13], who subjected coral skeletons with mature microbial euendolith communities dominated by the siphonous green alga *Ostreobium* sp. to present-day (400 ppmv) and future CO_2_ conditions (750 ppmv) for a period of three months. Microborers extended 40% further into their substrate in the high CO_2_ treatment, translating into a 48% higher dissolution rate. Reyes-Nivia *et al*. [15] exposed microborers occupying newly dead skeletons from two species of coral (*Porites cylindrica* and *Isopora cuneata*) to present-day and two future warming and OA scenarios. Microborers responded positively to treatments but differently within each substrate, resulting in an 89% and 45% increase in erosion for *P*. *cylindrica* and *I*. *cuneata*, respectively, under +610 μatm and +4 deg C. This response was observed only in illuminated samples, suggesting that it was primarily due to photosynthetic microborers such as chlorophytes within the genus *Ostreobium*, which usually dominate microboring communities in living corals [33, 34]. It is therefore clear that the effect of CO_2_ enrichment is variable and dependent upon complex combinations of drivers, such as environmental conditions and the density of the carbonate being infiltrated.

While these studies demonstrate a relationship between OA and increased rates of carbonate dissolution by microborers, controlled laboratory experiments often do not adequately replicate natural variability inherent in real-world systems. In addition, laboratory experiments are limited in that organisms may not be fully acclimated to treatment conditions. Furthermore, because laboratory work is confined to select taxa, it can miss the indirect effects of OA from multispecies interactions. For instance, high CO_2_ may enhance epilithic algal populations [9, 10] that can, in turn, influence microborer erosion rates [30]. Grazing sea urchins may also be influenced by OA [35], which may alter erosion rates and thereby indirectly influence the contribution of microborers to coral reef carbonate budgets [36]. To address these concerns and to study the effects of OA on natural, complex multi-taxa systems, work has been conducted on mesocosms [37], as well as in present-day ecosystems which naturally experience high pCO_2_.

It is interesting to note that most of the *in situ* studies on bioerosion under various pH conditions have concerned macrobioerosion of living corals while internal bioerosion (both micro and macroboring) is much more pronounced on dead skeletal materials, where coral tissues are not present to prevent the settlement of eroders [5, 23]. We are aware of only one study that has investigated the bioerosion of dead coral substrates as a function of seawater pH in the field. Silbiger *et al*. [17] deployed clean coral blocks along a 32 m transect in Kaneohe Bay for one year. Using microCT to quantify fine-scale changes in block volume (100 μm voxels), they found that erosion due to grazers and macroborers was more correlated with small-scale differences in pH than with other environmental parameters, including depth, distance from shore, nutrients, and temperature.

Despite investigation of grazing and macrobioerosion within numerous high pCO_2_ coral reef environments, we are unaware of any real-world corroboration of OA-enhanced bioerosion by microborers at reef sites experiencing projected pCO_2_ levels associated with OA. Using homogeneous calcium carbonate substrates previously employed for consistent, high-accuracy, and short-term quantification of boring taxa (e.g., [24]), we examine the settlement and erosion by microbial euendoliths at a volcanically acidified reef in the Commonwealth of the Northern Mariana Islands (CNMI). This represents the first study specifically targeting the colonization of a single erosive agent, i.e. microborers, in a natural reef environment mimicking carbonate conditions projected to occur by the end of the century due to OA.

## Materials and Methods

**Fig 1:**
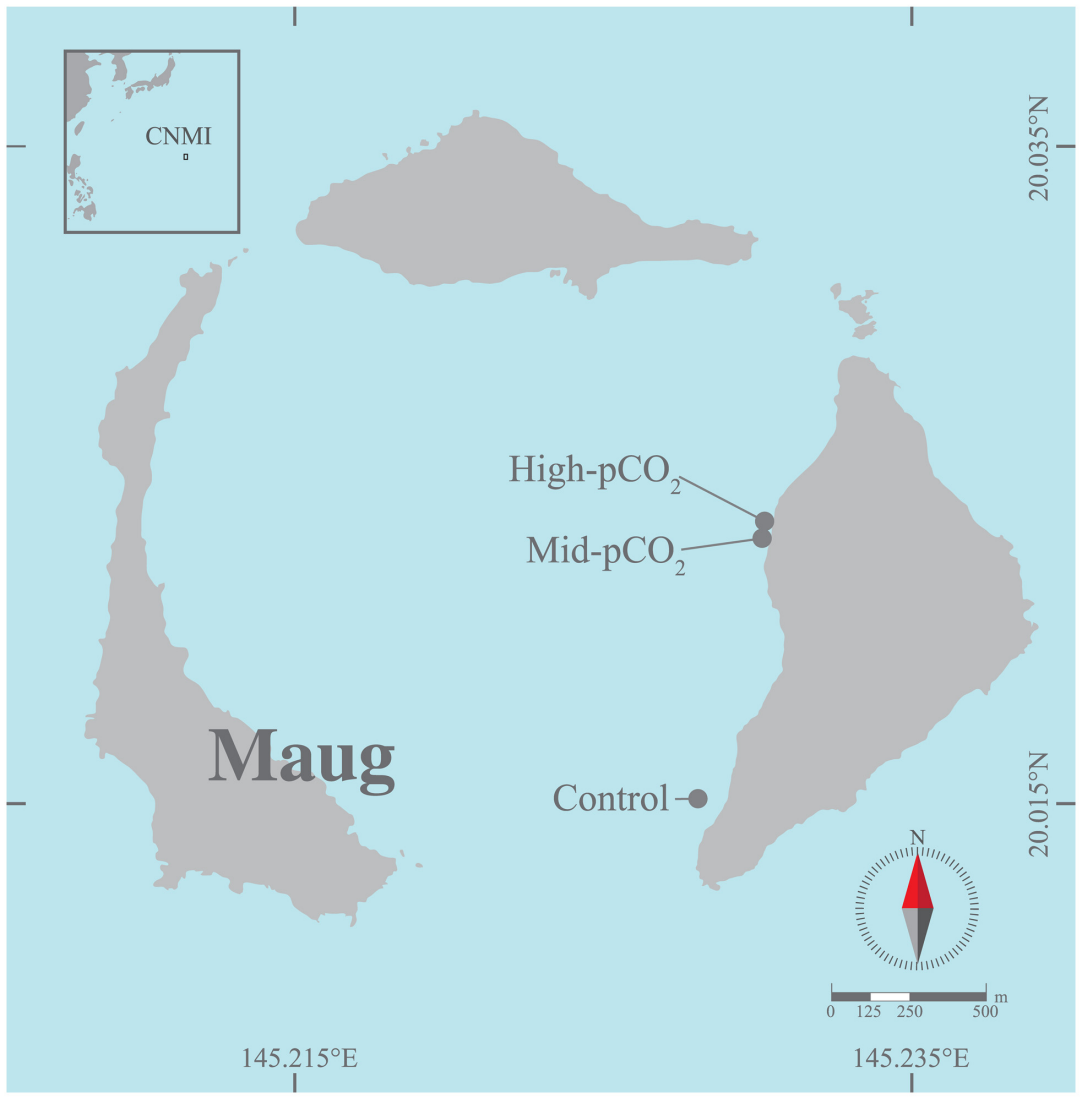
Map of Maug showing the locations of the High-pCO_2_, Mid-pCO_2_ and Control sites. CNMI, Commonwealth of the Northern Mariana Islands.

Maug Island (20°1'N, 145°13E) is part of CNMI and is located approximately 530 km north of Saipan (Fig 1). It is made up of three closely spaced islands, which are emergent parts of a volcanic caldera. Volcanic vents on the inner margin of the eastern most island locally elevate seawater pCO_2_ and impact benthic community structure [38].

Three study sites were selected spanning a gradient of vent-altered carbonate chemistry. A high-pCO_2_ site was established near the vent, though sufficiently far from the area of active bubbling so as to avoid complications with carbonate undersaturation (i.e. Ω < 1) and minimize the potential influence of other chemicals of volcanic origin. A mid-pCO_2_ site was established approximately 50 m to the south of the high-pCO_2_ site and a control site (with ambient pCO_2_) was located at the southernmost end of the island, approximately 1 km away. All sites were of similar depth (~9 m) and were instrumented with SeaFET pH and temperature (HOBO Water Temp Pro v2, Onset) loggers during the period of May 19 to August 10, 2014 as described in Enochs *et al*. [38]. Photosynthetically active radiation (PAR) loggers (ECO-PAR, WET Labs) collected data at the high and mid-pCO2 sites. Acoustic Doppler current profilers (ADCPs, Aquadopp, Nortek) were deployed at the high-pCO2 and control sites. Water samples were collected every six hours over a 48-hour period (Aug. 11–13 2014) immediately above the benthos and were analyzed for dissolved inorganic carbon (DIC) and total alkalinity (TA) following Enochs *et al*. [38]. Sampling dates and the duration of this experiment were constrained by limited access to the remote research site. All work presented herein was conducted in accordance with applicable rules and regulations governing fieldwork and sample collection at the study site. Research at the study sites was approved by the CNMI Department of Fish and Wildlife and microboring algae collected during this study are not protected species.

Blocks of clean unbored crystalline calcite (~2 x 2 x 1 cm, Icelandic spar) were affixed to grey PVC plates using JB-weld quick setting epoxy on the downward facing surface of the block. Eight blocks were attached in a line to PVC bases (~20 x 40 cm) per site, positioned flush to bare substrate, and deployed from May 17 to August 11, 2014. Blocks that came loose during deployment were not included in the analysis (n = 1, 1, 2, high-pCO_2_, mid-pCO_2_, control, respectively). Calcite blocks were air dried upon collection and transported back to Miami for subsequent analysis. They were then gently separated from their PVC bases and soaked in a 15% diluted hydrogen peroxide solution for two hours to remove non-calcified organics [24]. Each block was immediately rinsed and dried at 60°C for 24 hours.

Measurements were taken in order to quantify the degree of colonization or the percent planar surface area of microborers. Five haphazardly-placed images (~0.014 cm^2^ each) were captured from the upward-facing surface of each block (~2% block cover total) using a Motic BA310 light microscope at 100x magnification with the Motic Images Plus 2.0 software package. Magnification and replication were selected in order to maximize coverage while minimizing the potential for image overlap. Following capture, images were binarized to identify boreholes and subsequently their percent planar surface area was quantified using ImageJ [39]. This measurement was inclusive of boreholes both on and within the transparent surface of the block, though vacated (more clear) boreholes were likely under-represented. Values were expressed as percent coverage of each sample image. To measure depth of penetration of microborings (boreholes), spar blocks were split by fracturing along their vertical axis. Depth was measured using the same Motic microscope and software package as used with surface area measurements. Thirty depth measurements (length of surface to max filament depth) were obtained per block at haphazardly spaced points.

It was not possible to simultaneously measure the depth of penetration of a single borehole and its surface area at the block surface. Additionally, the total number of depth (30) and percent surface area measurements (5) were different. To address these issues and estimate erosion, thirty samples of percent surface area (to match borehole depth sample size) were generated per block by Monte Carlo sampling. This sampling was performed on truncated [0,100] gamma distributions [x1], which were created assuming sample mean and variance estimates of surface area were unbiased estimators of surface area covered by microborers on each block. Following, each randomly resampled percent surface area measurement was multiplied by a randomly selected depth measurement, resulting in 30 independent estimates of erosion (volume per unit surface area) for each block. Calculated standard errors of the erosion estimates therefore took into account the variability of both depth of borehole penetration and percent surface area colonized by microborers. Volumes of substrate eroded (per unit surface area) were then multiplied by the density of crystalline calcite (2.71 g cm^-3^) and then divided by the number of days the blocks were deployed in order to calculate bioerosion rate estimates for each block (kg m^-2^ yr^-1^).

Differences between sites and blocks were tested with Generalized Linear Models (GLMs). Blocks were nested within sites due to unknown, yet possible inherent characteristics within each spar block or from their orientation. For the GLM, a gamma distribution and log link function were employed, based on the distributions of the percent planar surface area and borehole depth data, as well as the resulting erosion rate estimates. Posthoc Tukey HSD tests were performed to identify pair-wise site and block differences. Post-hoc block comparisons were only conducted on blocks from a given site as blocks were nested within site treatments in the GLM model. All statistics were performed within the R Computing Environment [40].

## Results

**Fig 2:**
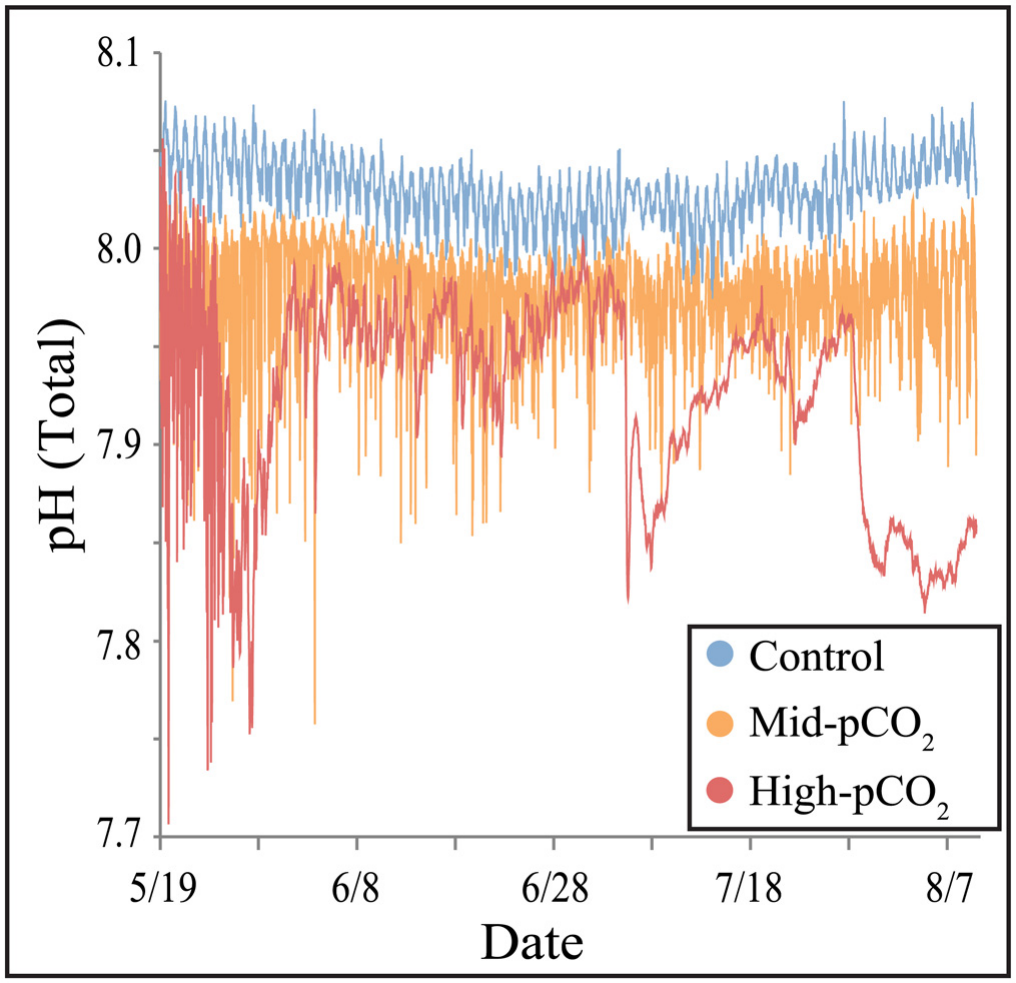
Seawater pH at each of the three experimental sites during the deployment of calcite blocks. Data are from Enochs *et al*. [38].

There was substantial depression of seawater pH in close proximity to the Maug CO_2_ vent, with successively higher pH observed at sites further away (Fig 2, Table 1). Data in Enochs *et al*. [38] show that this significant difference was due to localized pCO_2_ enrichment strongly influencing DIC despite relatively constant TA (Table 1). While other environmental variables differed among study sites (Table 1), differences were likely not of sufficient magnitude to be the dominant agents affecting community composition [38].

**Table 1:** Environmental data from the Maug study sites. Data and methods can be found within Enochs *et al*. [38]. Long-term data taken from multi-month deployment of data loggers over the duration of the experiment. Discrete bottle samples (n = 8) taken every 6 hours over a two-day period. Numbers in parentheses are standard deviation for long-term data and standard error of the mean for discrete bottle samples.

	Long-term data				Discrete bottle samples				
Site	pH (Total)	Temp (°C)	PAR (mol photons m^-2^)	Current (m s^-1^)	TCO_2_ (μmol kg^-1^)	TA (μequiv kg^-1^)	pH (Total)	pCO_2_ (μatm)	Ω_Arag_
**High-pCO**_**2**_	7.94 (0.051)	30.3 (0.98)	NA	0.09 (0.037)	1992.7 (10.93)	2283.4 (1.45)	7.95 (0.019)	502.0 (29.67)	3.4 (0.11)
**Mid-pCO**_**2**_	7.98 (0.027)	30.1 (0.99)	9.5 (2.46)	NA	1964.6 (10.44)	2281.4 (1.71)	7.99 (0.016)	441.2 (21.32)	3.7 (0.10)
**Control**	8.04 (0.016)	30.1 (0.98)	10.9 (2.46)	0.06 (0.023)	1944.8 (2.90)	2279.4 (1.61)	8.02 0.004)	401.3 (4.61)	3.8 (0.03)

**Fig 3:**
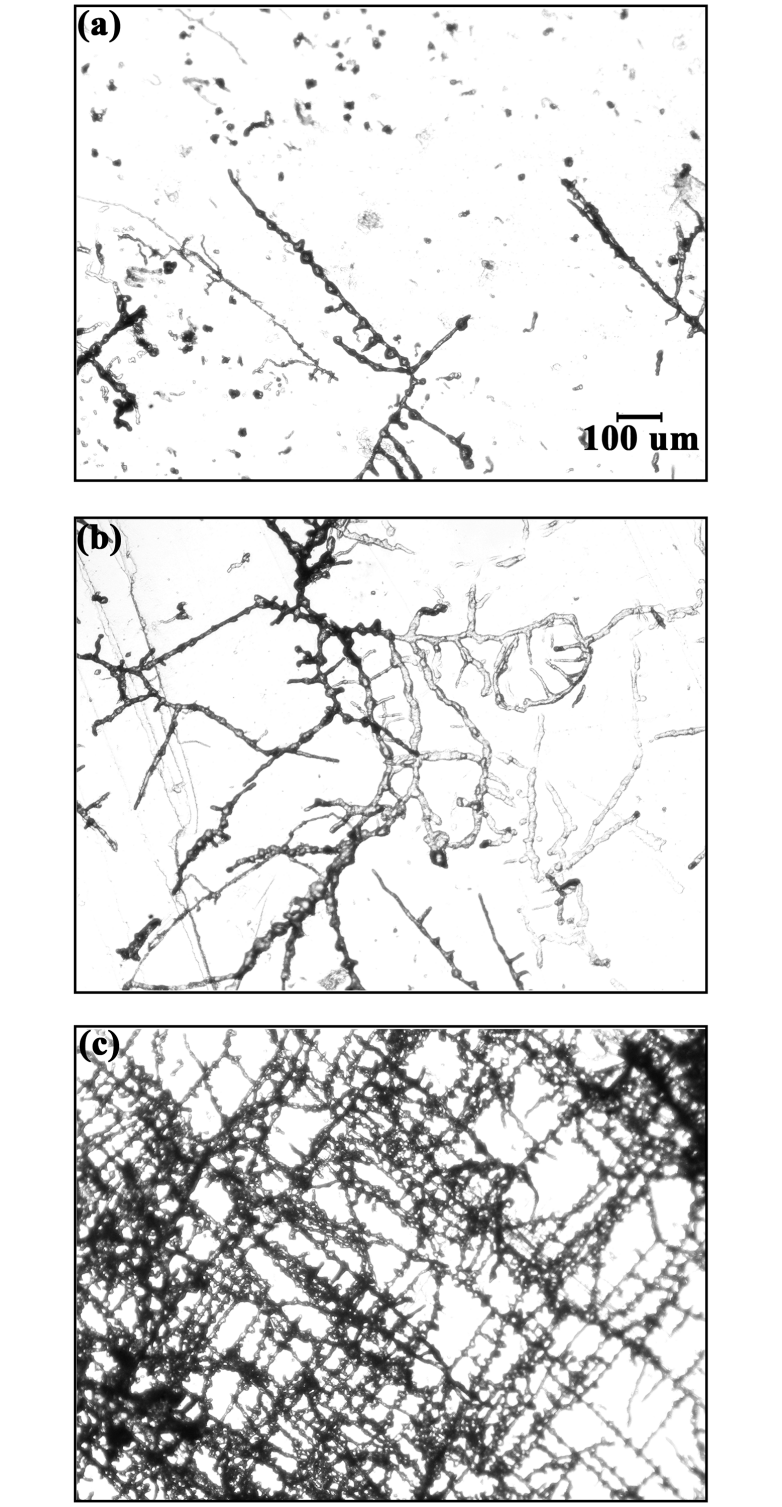
Top-down view of microborers within crystalline calcite at the Control (a), Mid-pCO_2_ (b), and High-pCO_2_ (c) sites. Scale bar in A is the same for all panels (100 μm). Images are the same size and magnification as those used for surface area analysis.

Qualitatively, blocks at the high-pCO_2_ site were less fouled with epilithic flora than at the mid-pCO_2_ and control sites, where turf algae had recruited during deployment. Microscopic analysis of spar revealed boreholes within all blocks at all sites, creating characteristic branching networks that spread laterally and did not penetrate greatly into the substrate (Fig 3). While species level identification was difficult given that spar blocks were dried, visual analysis of borehole morphology showed that microborer communities were relatively depauperate after three months of colonization and were dominated by chlorophytes commonly present in early successional stages of microboring assemblages, such as *Eugomontia* and *Phaeophila* [28]. Characteristic traces of *Rhopalia catenata* Radtke, 1991 were dominant, resulting from the activity of *Phaeophila* (see description of traces in Wisshak *et al*. [41]). Qualitatively, differences in community composition were not discernable among sites. Some very large unidentified filaments were also observed but were very rare.

**Fig 4:**
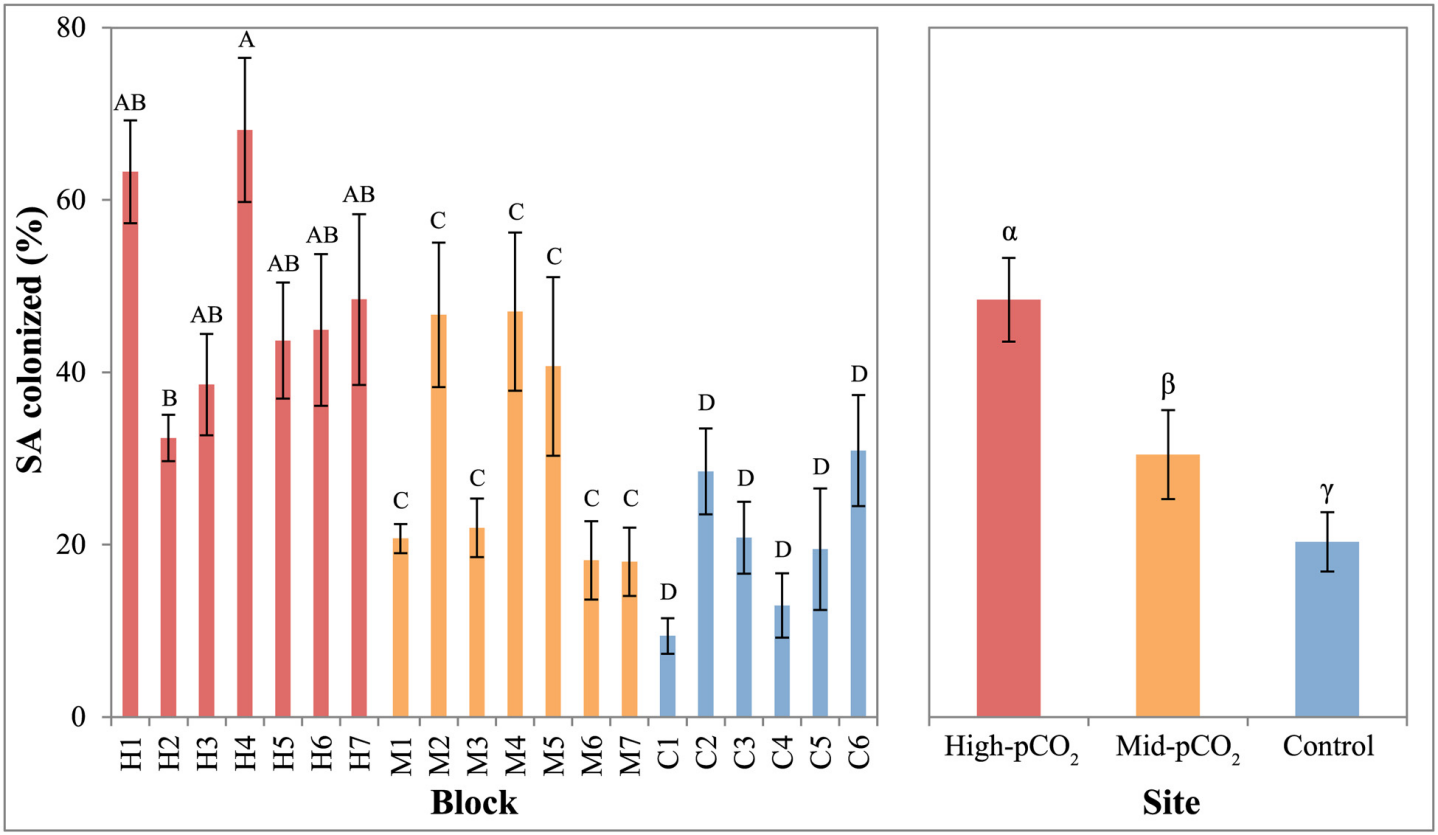
Mean percent surface area colonized by microborers in each block and between sites. Blocks within sites or sites that share a letter (Roman or Greek, respectively) are not significantly different (P>0.05). Error bars are standard error of the mean, calculated among individual measurements within each block (5 measurements per blocks, 6–7 blocks per site) and among all measurements within each site (30–35) in the left and right panels, respectively.

**Fig 5:**
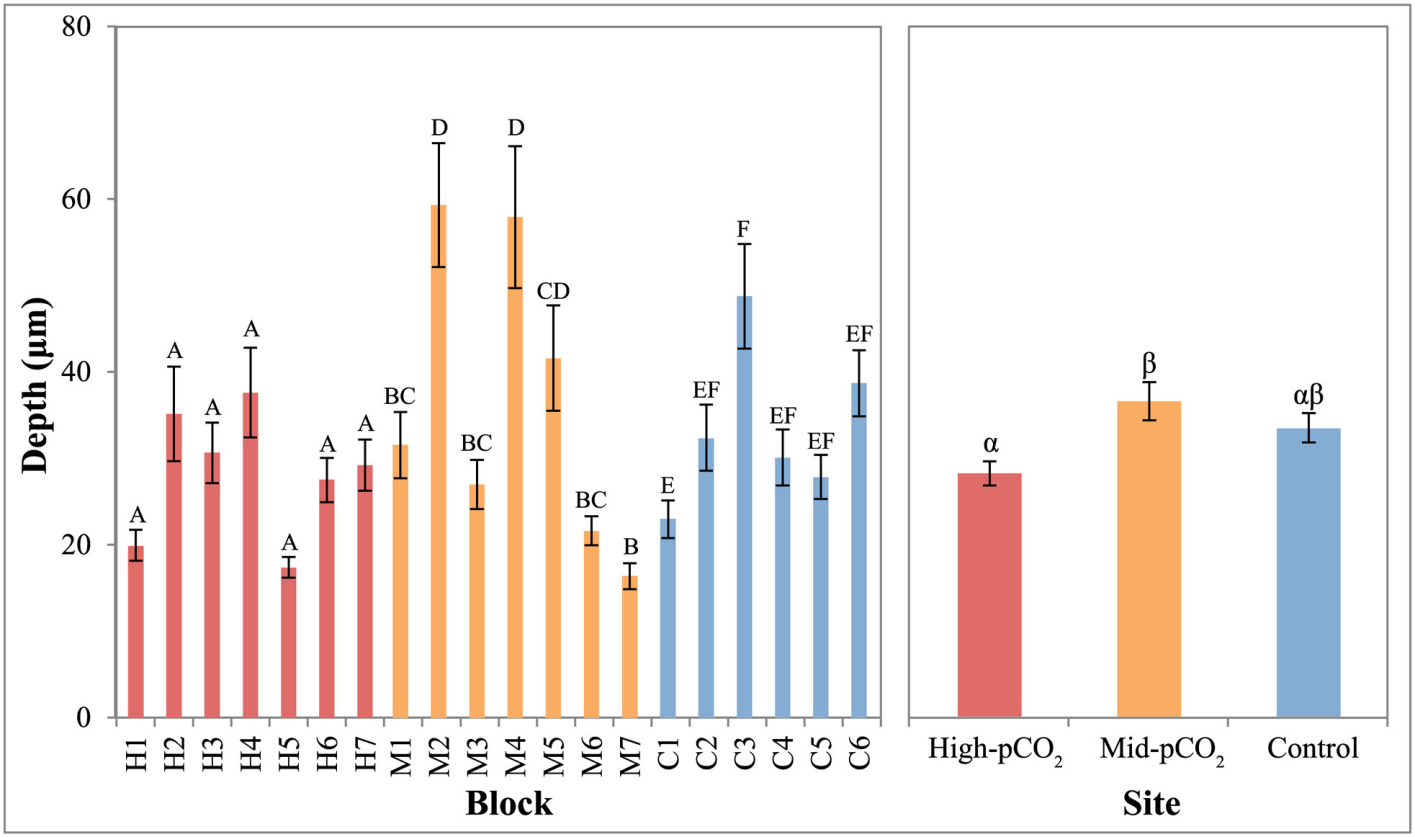
Mean depth of penetration of microboring filaments in each block and between sites. Blocks within sites or sites that share a letter (Roman or Greek, respectively) are not significantly different (P>0.05). Error bars are standard error of the mean, calculated among individual measurements within each block (30 measurements per block, 6–7 blocks per site) and among all measurements within each site (180–210) in the left and right panels, respectively.

**Fig 6:**
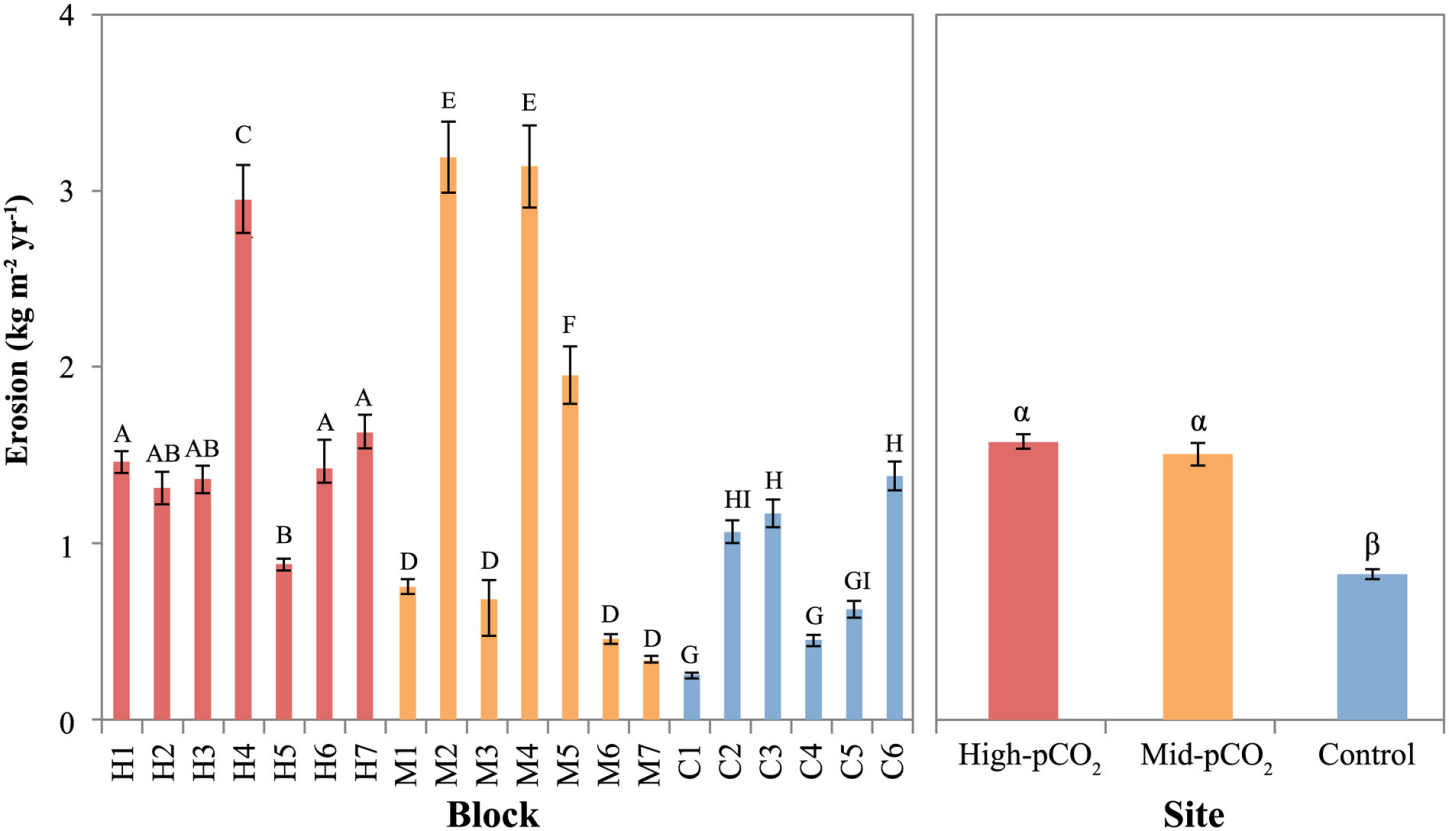
Mean rates of calcite erosion (mass loss) by microborers in each block and between sites. Blocks within sites or sites that share a letter (Roman or Greek, respectively) are not significantly different (P>0.05). Error bars are standard error of the mean, calculated among individual estimates within each block (30 estimates per block, 6–7 blocks per site) and among all estimates within each site (180–210) in the left and right panels, respectively.

The percent planar surface area of the entire microborer community was significantly different among sites (P<0.001), with cover increasing from control (20.3% ± 2.35, mean ± SEM) to mid-pCO_2_ (30.5% ± 3.15) to high-pCO_2_ sites (48.5% ± 3.22, Figs 3 and 4, Table 2). There was also a significant difference among sites for the vertical penetration of microborers into the blocks (Fig 5, Table 2). However, while the depth of microborer penetration was significantly higher at the mid-pCO_2_ (36.6 μm ± 2.20) than at the high-pCO_2_ site (28.3 μm ± 1.41), the control site (33.6 μm ± 1.66) was not significantly different than either. Microbioerosion rate, as calculated from horizontal extent and mean depth of penetration, was significantly greater at the high and mid-pCO_2_ sites (1.52 kg m^-2^ yr^-1^± 0.087, 1.46 ± 0.138, respectively) compared with the control (0.83 ± 0.060), though there were no significant differences between the two vent-influenced sites (Fig 6, Table 2).

**Table 2:** Analysis of deviance table from GLM of microborer colonization of calcite blocks at each of the three study sites. Df, degrees of freedom; Resid. Df, Residual degrees of freedom; Resid. Dev., Residual deviance.

Factor/source	Df	Deviance	Resid. Df	Resid. Dev.	F	Pr(>F)
**Surface area**						
Site	2	12.11	97	32.75	29.84	<0.001
Block(Site)	17	13.02	80	19.73	3.78	<0.001
Total residuals			99	44.86		
**Depth**						
Site	2	7.12	597	259.26	9.48	<0.001
Block(Site)	17	13.02	580	193.91	10.24	<0.001
Total residuals			599	266.37		
**Erosion**						
Site	2	39.38	597	535.94	35.18	<0.001
Block(Site)	17	236.84	580	299.10	24.89	<0.001
Total residuals			599	575.32		

There were significant sample effects within each site for all microborer parameters (Figs 4–6, Table 2), despite the deployment of homogeneous substrates, reflecting the patchiness of microborer settlement on benthic substrates. This was especially apparent in the microboring at the mid-pCO_2_ site, which exhibited a bimodal distribution among blocks (Figs 4–6). This pattern was evident in both the percent surface area colonized and depth of penetration of boreholes, translating into the calculated volume eroded as well.

## Discussion

Microborer communities that colonized calcite blocks after three months of exposure in this experiment were similar to those observed in previous reef studies on dead coral skeletons, reflecting that microborers studied at Maug were typical for reef environments and were not anomalous extremophiles. For instance, chlorophytes of the genus *Phaeophila* are typically dominant in early communities of microborers in reef carbonate substrates [28, 36, 42].

Higher colonization of block surfaces (planar surface area) by microborers in elevated pCO_2_ environments reported herein is consistent with previously published experiments that have shown a positive correlation between microborer community development and seawater pCO_2_ [13, 15]. However, in contrast to our findings, Tribollet *et al*. [13] did not record a significant increase in the percent planar surface area of boring algae. This suggests that immature microboring communities dominated by large pioneer chlorophytes such as *Phaeophila* sp. may respond differently to OA than mature communities dominated by the chlorophyte *Ostreobium* sp. Our study is unique in that we were able to use uncolonized substrates to examine short-term settlement, whereas previous experimental work applied acidification treatments to coral skeletons which had already been colonized by microborers (e.g., 6 mo. initial colonization, [13]). Our findings therefore pertain more closely to initial colonization, suggesting more rapid settlement following the fresh availability of carbonate, rather than expansion of established communities. In other words, surfaces were not 'saturated' by microborer communities during our investigation, allowing a clean-slate examination of differences in colonization among sites.

Tribollet *et al*. [13] and Reyes-Nivia *et al*. [15] measured higher rates of dissolution under elevated CO_2_ levels. Our data support these results. While the two vent-influenced sites in our study experienced higher rates of biogenic dissolution relative to the control site, they were not significantly different from each other. The strong difference in biogenic dissolution near the vent versus the control site is striking given the moderate levels of OA stress reflected by the mean pH of the mid- and high-pCO_2_ site (Table 1). Vent alteration of carbonate chemistry was variable throughout substrate deployment (Fig 2) and it is possible that exceptionally high microborer expansion/settlement occurred during periods of extreme pH depression. Further examination of the influence of temporal carbonate chemistry dynamics on microboring is necessary.

While the duration of our study (three months) was similar to previous experiments (three and two months, [13] and [15], respectively), these studies are not necessarily comparable to ours as they were conducted on more mature microboring communities inhabiting natural substrates. In our study, microborers would have had to both settle and proliferate during the duration of the experiment, rather than simply continue to erode and mature. Reyes-Nivia *et al*. [15] directly measured changes in weight, while we quantified the volume of CaCO_3_ removed by microborers using a similar microscopy technique to that used by Tribollet *et al*. [13]. The higher erosion rate observed in the Tribollet *et al*. [13] study was a result of deeper penetration of the carbonate substrate, rather than lateral proliferation. Given the aerial extent of the microborers in our experiment, we can reasonably expect that this same pattern would hold true in our study, had microborers had more time to establish stable climax communities (~1–2 yrs.) [28, 43]. Instead, the depth of penetration in our colonization study was highly variable and much lower than in mature communities studied in Tribollet *et al*. [13] (10’s μm vs mm).

Differences in depth of penetration among studies are likely due in part to the fact that the *Ostreobium* filaments in Tribollet *et al*. [13] are known to penetrate relatively deep into substrates (mm) as they are sciaphilic. By contrast, pioneer microborers such as *Phaeophila*, are photophilic microorganisms and usually stay within the first few hundred microns, boring parallel to the substrate surface and extending ‘rhizoidal’ appendages connecting the main algal cells to the external environment (water column) [22, 28]. We cannot rule out the possibility that the unique structure and transparency of the Icelandic spar substrate impacted an OA-related response in the depth of microborer penetration. Coral skeletons contain natural voids, pores, and fissures, which are utilized by microborers as they penetrate into the skeleton. These pathways are absent in the crystalline calcium carbonate utilized in this experiment, though higher transparency would have likely led to greater light availability within the substrate. Further, spar has a different crystalline structure (calcite) than coral skeletons (aragonite), which would yield different dissolution kinetics [44]. While some have concluded that substrate minerology has no influence on the rate at which they are bioeroded (e.g., clionaid sponges [45]), these differences may have nonetheless limited our ability to quantify a natural boring depth response and cannot be ignored when applying these data and trends to reef framework substrates.

While this is the first study to investigate the influence of a natural OA gradient on microborers, our findings are consistent with those that have examined correlations between macroboring intensity and seawater pH over spatial scales ranging from meters to thousands of kilometers. Silbiger *et al*. [17] demonstrated a positive relationship between seawater acidity and coral skeleton erosion resulting from grazing and macroboring across small spatial scales (≤ 32 m) on a Hawaiian reef. The use of high-resolution microCT allowed unprecedented detection of small boring animals (e.g., polychaetes); however, the 100 μm resolution was too coarse to resolve microborer networks. Similarly, both DeCarlo *et al*. [46] and Barkley *et al*. [47] used CT to show that pH was negatively correlated with macrobioerosion in skeletons of living massive *Porites*, though the resolution of their analysis was only successful at quantifying voids greater than 1 mm diameter. Further work is therefore needed to accurately measure bioerosion by microborers along natural CO_2_ gradients.

Accelerated dissolution of carbonates by microborers may act to enhance both macroboring and grazing. Reef bioerosion undergoes a successional pattern, starting with the initial settlement and penetration of microborers. This is followed by relatively small macroboring fauna, such as polychaetes, which create boreholes that are subsequently colonized by larger macroboring taxa such as bivalve mollusks [23, 48, 49]. Accelerating initial microborer colonization could result in more rapid advancement through these successional stages. Furthermore, enhanced dissolution by microbial euendoliths could weaken substrates, potentially facilitating the erosion of macroboring fauna. euendolithic algae are an important nutrient source for grazing animals such as parrotfish, and more-porous substrates weakened by microborers may be easier to break and scrape [50]. As such, higher abundances of microborers may also be correlated with greater grazing [29].

As with studies in other natural systems, it is impossible to completely eliminate covarying environmental factors that could influence settlement of microborers. Enochs *et al*. [38] measured lower light intensities closer to the Maug vent site (~13% lower, control vs. mid-pCO_2_ site, Table 1). Reduced light availability can limit the depth of penetration of photosynthetic microborers into coral substrates (reviewed in [22]), alter photophysiology [51], and may reduce settlement in low light environments [52]. Light limitation could have therefore resulted in lower colonization near the vent, reducing the CO_2_-enhanced colonization signal. However, given the transparent nature of the substrates used in this study, the depth at which the experiment was carried out, and the low light requirements of photosynthetic microborers, the slight depression in light near the vent should have had a minimal impact [22, 51].

The strong relationship between macroboring intensity and water quality is well represented in the literature, where numerous endolithic fauna are suspension feeders and rely on planktonic organic matter [49, 53]. The influence of water quality on microboring communities, however, is not as well understood. Kiene [42] found no significant difference in the colonization of endolithic microborers after a five-month nitrogen and phosphorous enrichment experiment on the Great Barrier Reef. By contrast, in a 49-day experiment conducted on molluscan shell substrates in Belize, Carreiro-Silva *et al*. [31] observed significant enhancement of phototrophic microborers under elevated inorganic nutrient enrichment and increased populations of heterotrophic microborers under enriched organic matter. Other water quality parameters such as sedimentation, however, are also known to negatively influence microborer proliferation [29]. On a large scale, Maug Island exists in the remote Mariana Islands, far removed from anthropogenic nutrient enrichment and plumes of terrigenous sediments. Further work is needed to quantify nutrient and sediment concentrations at Maug, over both localized and regional scales, in order to determine the extent to which they directly influence the patterns observed in this study.

While steps have been taken to eliminate extraneous sources of variance in previous studies (e.g., herbivore exclusion, [31]) there is evidence that indirect interactions may play a role in mediating the relationship between nutrients and microborers. For instance, Chazottes *et al*. [30] found that higher nutrient environments favored the development of macroalgae and crustose coralline algae, rather than turfs, effectively limiting the removal of microborers by large grazing fishes and urchins. Turf algae, which were abundant in less nutrient-rich environments, were actively grazed upon, effectively removing microborers within the upper layers of the carbonate substrate in their study. While food availability may have altered grazing pressure [54] at Maug, we do not expect it to have influenced patterns in microborer abundance, penetration, or dissolution measured herein. Experimental calcite blocks were still smooth after collection and there was no evidence of grazing scars, which would have removed microborers.

Enhanced total bioerosion (i.e. microboring + macroboring + grazing) may be partly responsible for the relative paucity of framework near the Maug vent. At the control site, which experienced the lowest settlement of microborers, large framework structures were abundant, including massive *Porites* corals several meters in diameter [38]. Reef framework was evident at the mid-pCO_2_ site but was qualitatively less developed than the control site. No carbonate framework was present near the vent, though several coral colonies were found in the area. Part of this pattern is likely due to decreased calcification or recruitment of corals, but OA-accelerated bioerosion may also play an important role as the absence of CaCO_3_ left behind from dead coral is indicative of poor preservation potential.

Enochs *et al*. [20] modeled the effects of accelerated erosion (macro and microboring) and reduced calcification on coral reefs in the Florida Keys and found that net carbonate budgets will become more negative (erosional) in increasingly acidic waters. This problem will be exacerbated by warming-induced coral bleaching and mortality [55]. While rates of external bioerosion may be relatively unaffected by coral mortality in some regions (e.g., [56]), in others, newly available bare substrates can be readily colonized by bioeroding flora and fauna, resulting in a unique succession of eroding taxa that can lead more rapidly to skeletal loss [34]. If the settlement and colonization of these newly accessible substrates by microborers is indeed accelerated by OA as we found at Maug, not only will this further tip the balance towards a net erosive state, but it will also decrease the time during which other encrusting organisms could colonize the substrate and inhibit the destructive action of erosive communities.

Further research is needed on biogenic carbonate dissolution by microborers in OA conditions, especially from natural systems where complex indirect interactions may enhance or hinder microborer community dynamics. Longer studies (3–5 yrs.) designed to specifically document the full successional progression from the initial pioneering microborer communities to the climax communities of a consortium of micro- and macroborers would be particularly instructive. Given previous experimental evidence suggesting accelerated dissolution under OA scenarios [13, 15] and increased settlement rates reported here, it is likely that microborer erosion will increase as atmospheric CO_2_ continues to rise. This will be further enhanced by the greater availability of bare substrates due to warming-related coral mortality [20, 55] and elevated microborer biomass in bleached corals [57]. Coupled with accelerated macrobioerosion [14, 20, 46, 47] and reduced calcification [7], the complexity of reef habitats can be expected to continue to decrease [58].
